# Melanotic neuroectodermal tumor of infancy (MNTI): A report of two cases from Pakistan

**DOI:** 10.12669/pjms.41.13(PINS-NNOS).13348

**Published:** 2025-12

**Authors:** Ahtesham Khizar, Sundas Irshad, Maryem Tanweer, Haseeb Mehmood Qadri, Saad Maroof Saeed, Hassaan Zahid

**Affiliations:** 1 Ahtesham Khizar, Punjab Institute of Neurosciences, Lahore, Pakistan; 2 Sundas Irshad, Punjab Institute of Neurosciences, Lahore, Pakistan; 3 Maryem Tanweer, Punjab Institute of Neurosciences, Lahore, Pakistan; 4 Haseeb Mehmood Qadri, Punjab Institute of Neurosciences, Lahore, Pakistan; 5 Saad Maroof Saeed, Shaukat Khanum Memorial Cancer Hospital-Outreach Laboratory, Faisalabad, Pakistan; 6 Hassaan Zahid. Punjab Institute of Neurosciences, Lahore, Pakistan

**Keywords:** Developing country, Melanotic neuroectodermal tumor, Neurosurgery, Skull neoplasms, Vanillylmandelic acid

## Abstract

Melanotic Neuroectodermal Tumor of Infancy (MNTI) is a rare tumor of neural crest origin seen in the pediatric population aged less than one year. Despite their very aggressive growth, they are mostly benign in nature, commonly involving the maxilla, but in very rare cases, the skull and brain have also been seen to be affected. We are presenting here two rare cases, including a six months old girl with right orbitotemporal swelling and a three months old boy with right temporal swelling. Both cases were presented consecutively with a gap of one month between April and May 2024 at the Punjab Institute of Neurosciences, Lahore, Pakistan. CT and MRI in both of these cases showed extra-axial lesions. Complete excision of the tumor in both cases was performed with no postoperative complications or recurrence of the tumor at six-month follow-up. The purpose of this case series is to describe these unusual cases of cranial MNTI in infants in order to gain useful insights into the clinical course, diagnostic complexities, and therapeutic techniques for cranial MNTI. This is the first case series on cranial MNTI to be reported from Pakistan.

***List of abbreviations:* CT:** Computed tomography, **MNTI:** Melanotic Neuroectodermal Tumor of Infancy, **MRI:** Magnetic resonance imaging, **VMA:** Vanillylmandelic acid

## INTRODUCTION

Melanotic Neuroectodermal Tumor of Infancy (MNTI), also known as pigmented epulis, retinal anlage tumor, melanotic progonoma, congenital melanocarcinoma, or melano ameloblastoma, is a rare, rapidly progressive, recurrent tumor of infants accounting for approximately 500 cases globally.^1^ MNTI are predominantly located in the maxilla, accounting for 70% of cases; however, Ren et al. documented 91 occurrences of cranial MNTI in their review of literature from the last ten years.^2^ Vanillylmandelic acid (VMA) levels, though occasionally elevated in MNTI, demonstrate limited diagnostic sensitivity, as evidenced by Ren et al., who reported positive results in only two of 18 preoperatively tested patients.^2^ Histologically, these tumors are identified by their biphasic cellular distribution of peripheral large epithelioid melanogenic cells and central small primitive neuroblasts, implying their origin from neural crest cells. Immunohistochemically, these tumors stain positive for HMB45, vimentin, neuron-specific enolase, cytokeratin, and synaptophysin.^3^

Patients with cranial MNTI exhibit a clinically significant, rapidly advancing, non-tender, firm mass located beneath the epithelial surface or elevated intracranial pressure if the mass is situated within the skull. Additionally, there are increased urinary levels of VMA, which decrease following the surgical removal of the tumor.^3^ Owing to the locally aggressive behavior of MNTI, the preferred treatment option is complete surgical excision with clear margins.^4^ The recurrence rates reaching up to 27% have been reported after complete surgical excision, while older age at diagnosis, larger size, conservative treatment, subtotal surgical resection, development of malignancy, and metastasis have been identified as key factors affecting the recurrence of cranial MNTI.^3^ The role of chemotherapy and radiotherapy, whether as standalone therapy or in conjunction with surgical treatment, is still controversial due to the potential risk of radiation exposure and drug toxicity in younger patients.^4^

To date, only one case report from Pakistan describing the rare occurrence of MNTI in an eight-month-old girl in the anterior parasagittal skull has been identified by Khan et al.^5^ This case series aims to document the two rare occurrences of cranial MNTI in three- and six-month-old infants to provide valuable insights into the clinical course, diagnostic intricacies, and management strategies for cranial MNTI. We present the following cases in accordance with the CARE guidelines.^6^

## CASE PRESENTATION

This is a retrospective conservative case series of two cases that were treated with a gap of one month between April and May 2024 at the Punjab Institute of Neurosciences, Lahore, Pakistan.

### Case-1:

A six months old girl presented with complaints of right orbital swelling gradually increasing in size for two months. There was no associated history of fever, cold, vomiting, weight loss, or epileptic seizures. Her immunization was up-to-date, and developmental milestones were also appropriate. On examination, there was a swelling involving the right orbitotemporal region, roughly 5 x 4 cm in size, no discoloration of the overlying skin, and no discharge. The temperature of overlying skin was normal; swelling was firm in consistency, non-tender, non-pulsatile, and attached to underlying structures, while overlying skin was mobile.

**Fig.1:**
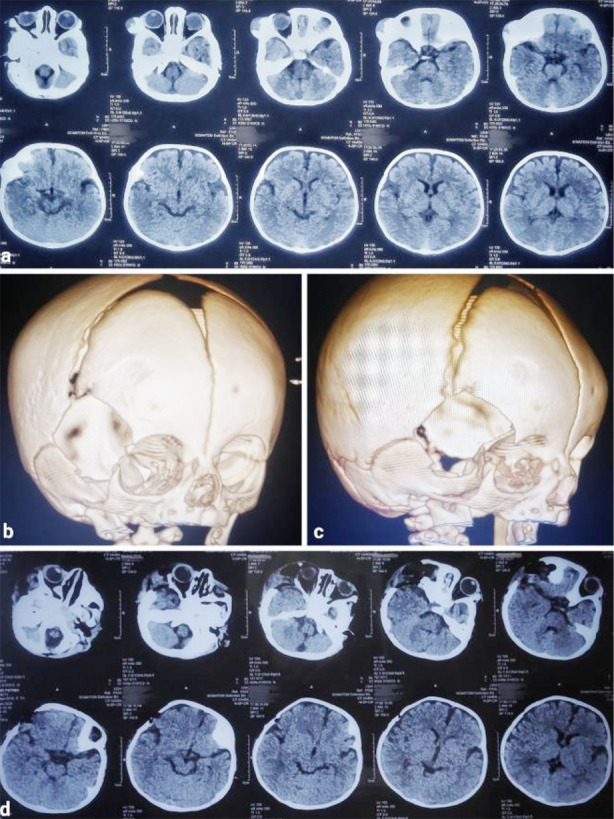
(a) Preoperative CT brain without contrast showing iso- to hypodense soft tissue swelling with surrounding hyperdense bone overgrowth of the lateral orbital wall and right temporal bone. (b & c) Postoperative CT with 3D skull reconstructions showing resection of the right frontotemporal bone and lateral orbital wall. (d) Postoperative CT brain without contrast showing complete resection.

On a computed tomography (CT) scan without contrast, an iso- to hypodense soft tissue swelling with surrounding hyperdense bone overgrowth of the lateral orbital wall and right temporal bone was seen along with bony spurs attached to the lesser wing of the sphenoid, measuring 48 x 43 x 14 mm in size (Fig.1: (a)). As the lesion was purely bony, magnetic resonance imaging (MRI) was not done, and surgery was performed on the basis of CT findings. Our initial diagnosis was osteoma, though it is uncommon in this age group.

**Fig.2:**
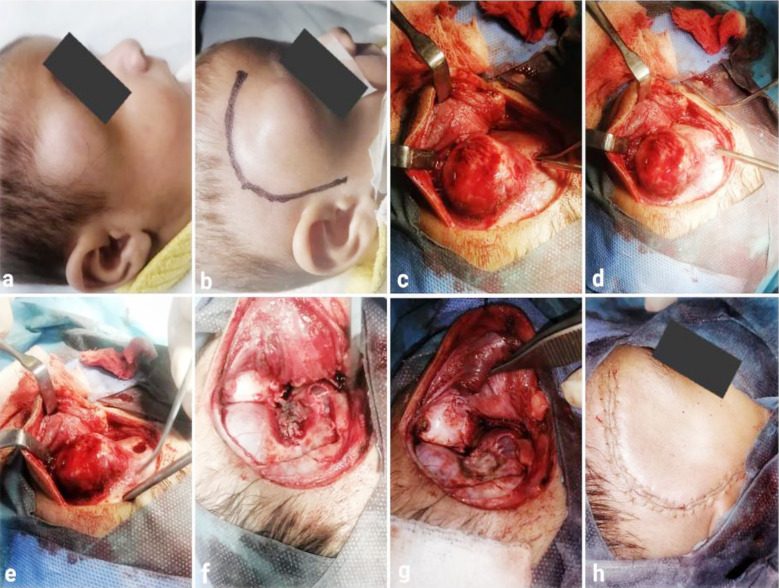
(a) Preoperative image of right orbitotemporal swelling. (b) Incision marking. (c & d) Complete exposure of lesion. (e) Single burr hole made. (f) Complete en bloc excision of involved bone. (g) Underlying dural attachment coagulated. h: Postoperative image after skin closure.

A right-sided curvilinear incision over the frontotemporal region in front of the ear was given, involved pericranium, and hyperostotic bone was completely excised via a single burr hole and bone cutting with a drill system. The dura was coagulated, as there was a slight attachment of the lesion to it (Fig.2: (a, b, c, d, e, f, g, & h)). Closure was done without performing cranioplasty, as it is not recommended in the pediatric population with growing skull bones. There were no intraoperative complications, and postoperative recovery from anesthesia was smooth and uneventful. The extent of bony resection can be seen in postoperative CT scans without contrast and 3D reconstructions, as shown in Fig.1: (b, c & d).

**Fig.3:**
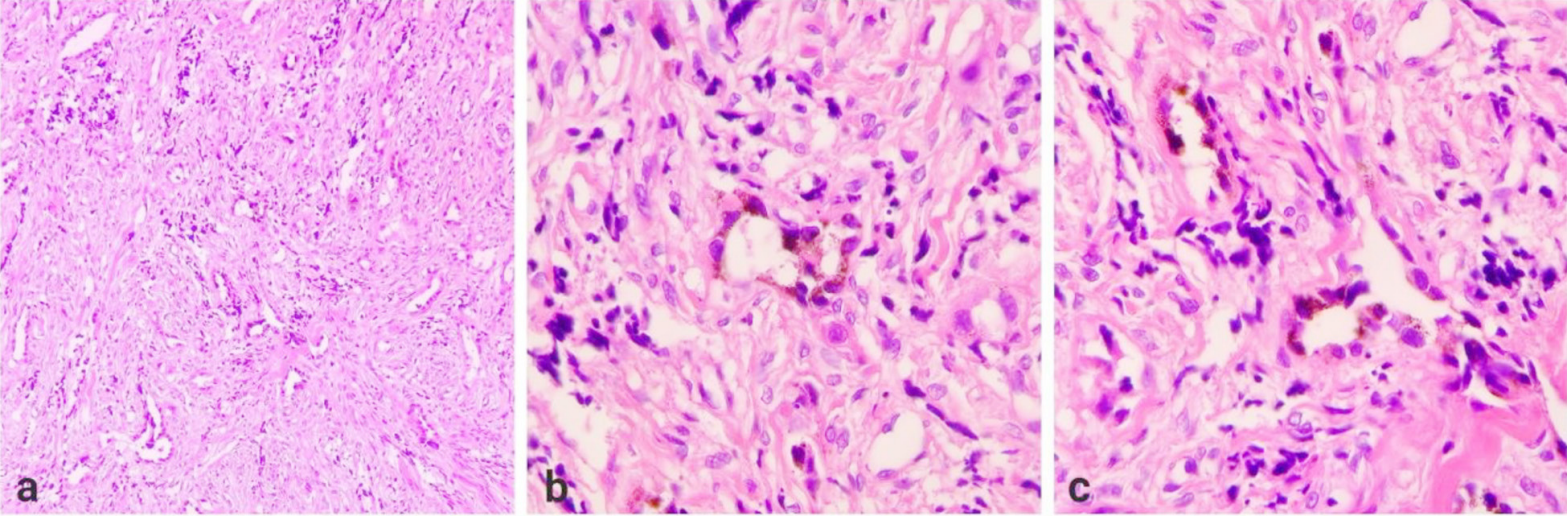
(a) Section showing a biphasic population of cells consisting of round blue cells with hyperchromatic nuclei and a second population with larger epithelioid cells arranged in nests.(100x). (b & c) Melanin pigmentation is seen.(400x).

Bony and soft tissue biopsy samples collectively measuring 50 x 45 x 15 mm were sent for histopathology, which showed a biphasic population of cells consisting of round blue cells with hyperchromatic nuclei and a second population of large epithelioid cells arranged in nests, thus confirming a diagnosis of MNTI as shown in Fig.3: (a, b, & c). On follow-up at six months, the child was asymptomatic with no cosmetic disfigurement, no complications or regrowth of the tumor, and milestones were normal for age.

### Case-2:

A three-month-old boy presented with a history of rapidly growing swelling over the right side of his temporal scalp for three weeks. He had no history of fever episodes, colds, vomiting, or seizures, and his weight (5.8 kg) was also adequate according to age. His scheduled immunization was up-to-date, and developmental milestones were also normal. On examination, there was a swelling involving the right temporal region measuring 3 x 2 cm in size; overlying skin was normal with no erythema and no discharge; on palpation, temperature was normal, non-pulsatile, firm in consistency, non-tender, and attached to underlying structures, while overlying skin was mobile.

**Fig.4:**
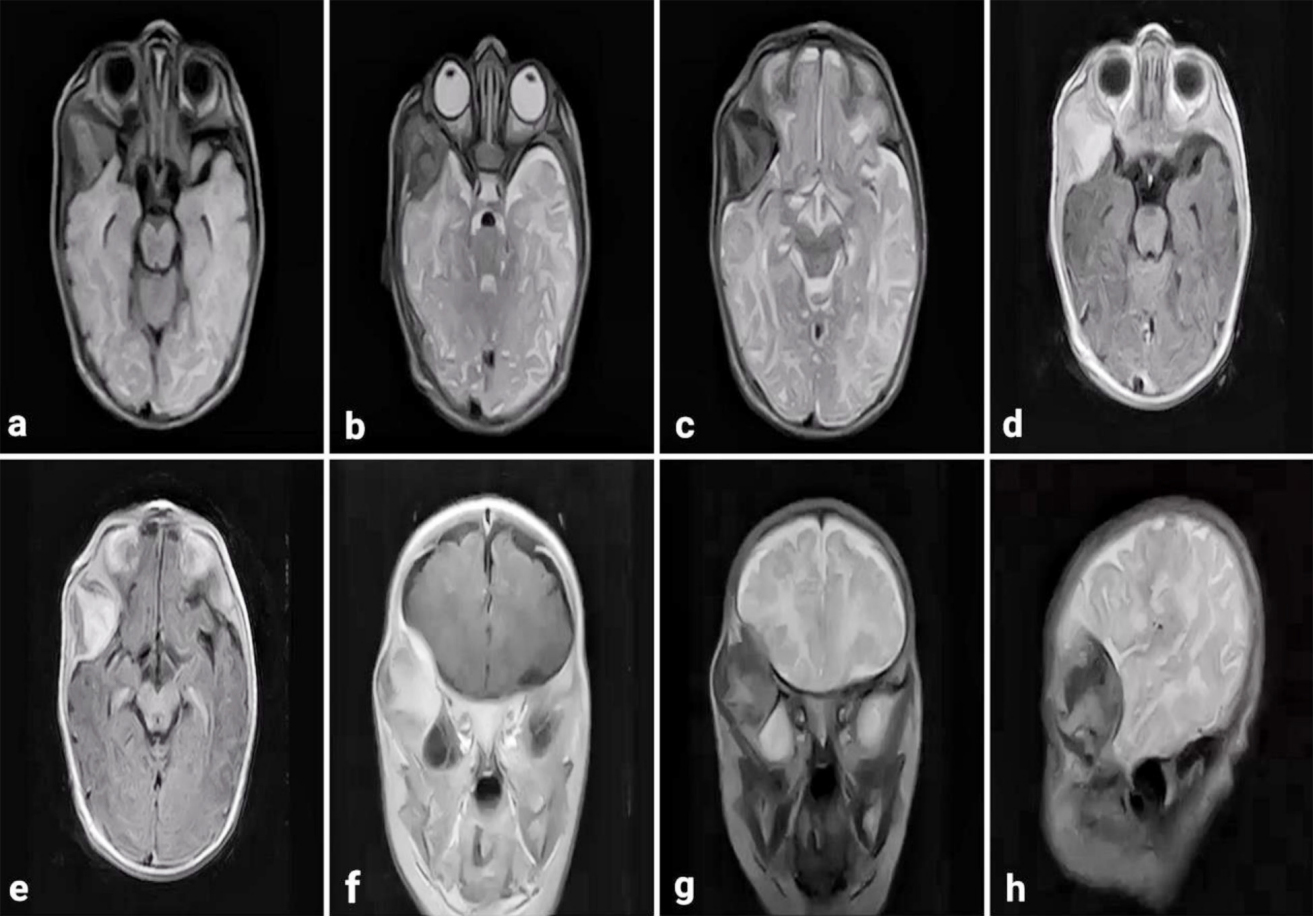
(a) MRI brain T1-weighted axial view showing a hypointense extra-axial right temporal lesion. (b & c) T2-weighted axial view showing a similar lesion. (d & e) T1-weighted with contrast axial view showing contrast enhancement of the lesion and dural attachment. (f) T1-weighted with contrast coronal view also showing contrast enhancement. (g) T2-weighted coronal view showing hypointense lesion in right temporal base. (h) T2-weighted sagittal view of the same lesion.

A CT scan without contrast showed a well-defined extra-axial lesion measuring 29.4 x 20.1 x 30 mm in the right temporal region, iso- to hyperdense, along with hyperostosis of the right temporal bone and greater wing of the sphenoid, causing widening of the adjacent coronal suture. There was obliteration of the right sylvian fissure and compression of the underlying temporal lobe. An MRI of the brain with and without contrast was done, which showed an extra-axial lesion 3.4 x 3 x 4.2 cm along the dural surface of the right temporal lobe, hypointense on T1, isointense on FLAIR, and hyperintense on T2, with avid contrast enhancement along with hyperostosis of adjacent temporal bone with expanded diploic space. (Figure. 4: a, b, c, d, e, f, g & h) So based on history, examination, and radiology, our differentials were meningioma and fibrous dysplasia.

**Fig.5:**
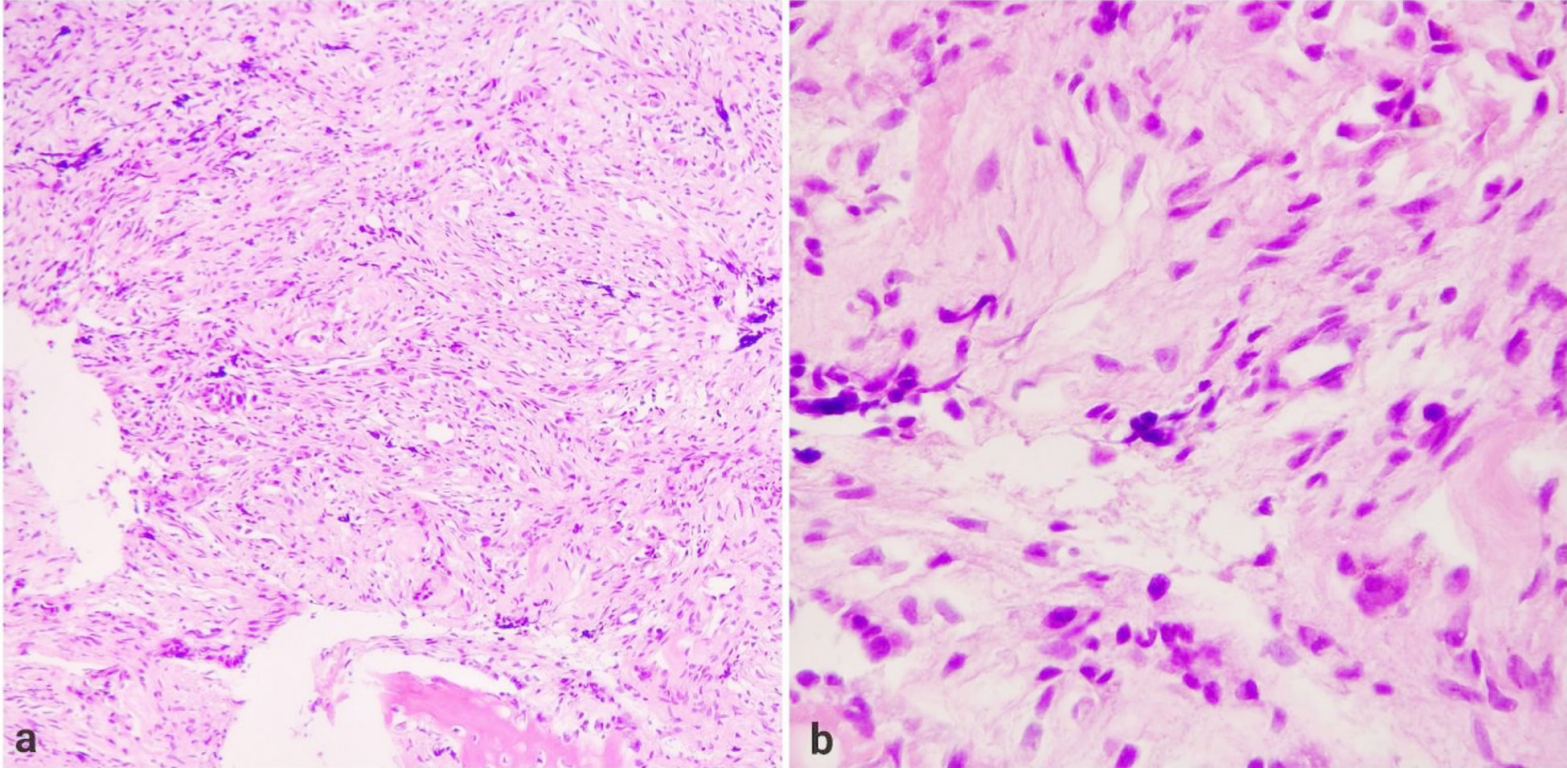
(a & b) Sections show a biphasic population of cells composed of nodules of round blue cells that are small and hyperchromatic. Other populations of larger epithelioid cells with melanin pigment are also seen.

Surgical wide local excision of the lesion was performed via a curvilinear incision given in front of the right ear; approximately 4.5 x 4 x 4.5 cm of thickened bone was removed along with soft grayish tissue attached with dura. Closure was done without any cranioplasty, as the patient was of growing age and the diagnosis was yet to be confirmed by histopathology. There were no intraoperative complications, and recovery from anesthesia was uneventful. Biopsy samples containing multiple bony and soft tissue fragments were sent for histopathology, which revealed a biphasic population of cells composed of nodules of round blue cells, small and hyperchromatic. Other populations showed large epithelioid cells with melanin pigment. Thus, on the basis of the histopathology report, the diagnosis of MNTI was confirmed, as shown in Fig.5 (a & b). The patient was doing fine with no recurrence, and no complications were observed on follow-up at six months, and milestones were also normal according to age.

## DISCUSSION

Melanotic Neuroectodermal Tumor of Infancy (MNTI) is a rare neoplasm that primarily affects infants with male predominance.^2^ This case series provides a description of two clinical cases of cranial MNTI, one male infant and one female infant, which suggests its occurrence in both genders. This is different compared to the previously reported literature, which indicated a higher male prevalence with a reported male-to-female ratio of 3:1.^7^ In the two cases presented in this case series, patients presented clinically at three and six months of age, respectively. This coincides with the median age range of one to six months identified in the literature by Xia et al.^8^ Similarly, it is well supported in the literature by the findings of a systematic review by Ren et al., who reported that approximately 70% of infants with skull MNTI are diagnosed within six months of their lives.^2^ Moreover, he found that 88.7% of patients with skull MNTI presented with rapidly growing, painless scalp swelling without neurological deficit, similar to the clinical presentation of cases discussed in our series.^2^

Radiologically, MNTI are recognized as isodense to hyperdense lesions on CT scans without contrast that are associated with hyperostosis or sclerosis of surrounding bones, as described in our cases.^9^ MR imaging of these lesions shows decreased signal intensity on T1-weighted images, hyperintensity on T2-weighted images, and strong contrast enhancement. This is contrary to the findings of Krawczyk et al., who proposed the occurrence of increased signal intensity on T1-weighted sequences of MR imaging in MNTI owing to chelation of paramagnetic metal ions by melanin.^9^ This variation in radiological picture might be attributed to the fact that MNTI may show variable radiological features depending upon the amount of melanin in tumors or variation in histopathological features with massive areas of calcification leading to decreased signal intensities.^9^,^10^ Based on the clinical presentation and radiological features of the bony lesions, our preoperative differentials were meningioma, osteoma, and fibrous dysplasia. Neuroblastoma and rhabdomyosarcoma are other common radiological differentials in this age group, but we ruled them out based on history and examination.^11^

Although urinary levels of vanillylmandelic acid (VMA) are reported to be raised in cases of MNTI,^9^ it was not done in our case owing to a lack of preoperative suspicion of this rare pathology. Yet, this is well supported by the findings of a systematic review of 91 cases of skull and brain MNTI in which Ren et al.^2^ stated that VMA levels were assessed in only 18 patients preoperatively and were found positive for only two patients.

The definitive treatment plan for MNTI, as discussed in literature, is complete surgical resection with clear margins, while the use of adjuvant chemotherapy is controversial.^2^ We did complete surgical resection of the tumor without immediate reconstruction due to the anticipated risk of recurrence. The biphasic cellular distribution of the tumor was confirmed in the histopathology of both cases, consistent with the findings of previously reported literature.^2^,^6^ The immunohistochemical analysis of MNTI is typically positive for all the markers of neural crest cell origin, i.e., HMB-45, NSE, and synaptophysin.^9^ However, it was not done in our cases owing to resource limitations.

Although recurrence rates of up to 30% have been reported in the literature,^2^,^6^ both patients in our case remained disease-free during the follow-up period, highlighting the effectiveness of early surgical intervention. However, longer follow-up is required to assess long-term outcomes.

This case series recommends the need for establishing a record of MNTI cases being diagnosed globally in order to develop a deep insight into the epidemiological trends and treatment outcomes of patients with long-term follow-up. Moreover, as the disease may need extensive excision, we should focus on developing optimal reconstructive techniques to limit functional impairment while simultaneously considering the potential recurrence patterns of the disease. Future research aimed at refining management strategies and enhancing patient outcomes, especially in low-resource settings like Pakistan, is recommended.

## CONCLUSION

MNTI is a rare, locally aggressive pediatric tumor. This case series is an effective addition to the growing body of literature while focusing on the involvement of the temporal bone. Although complete surgical resection of the tumor is the primary treatment for the disease, challenges associated with early diagnosis and postoperative treatment surveillance emphasize the importance of identifying this rare presentation as one of the differentials and planning structured follow-up programs.

### Authors’ Contributions:

**AK:** Concept and design of study, data acquisition, and manuscript writing.

**SI MT:** Data acquisition and manuscript writing.

**HMQ:** Data acquisition and critical review.

**SMS HZ:** Supervision and Critical review. Data interpretation and analysis.

All the authors have read and approved the final manuscript for the publication. All agreed to be accountable for all aspects of the work related to accuracy or integrity.
